# Illumination of the Cavities of the Body by a New Instrument—Nitsche

**Published:** 1879-09

**Authors:** A. Osterday


### TRANSLATIONS.

### ILLUMINATION OF THE CAVITIES OF THE BODY BY A NEW INSTRUMENT—NITSCHE.

TRANSLATED FROM THE GERMAN BY A. OSTERDAY, M. D.

NOT a little sensation is at present excited in surgical circles by the invention of a new illuminating apparatus, by which the surgeon is enabled to illuminate all cavities of the body accessible from the outside, as the bladder, rectum, stomach, etc., and inspect in such a manner that he may obtain a precise view of the internal condition of the illuminated cavities. Repeated experiments made by Prof. Dittel, in the presence of eminent surgeons, on living subjects, have proved the extraordinary merits of the invention. Hitherto this apparatus has been used for illumination of bladder, urethra and rectum, and has proved itself most excellent. One may see in the illuminated bladder the smallest piece of gravel, the smallest injected vessel. The operator has not to depend on his manipulations and his sense of touch; if he seeks for stone in the bladder, or treats any other vesical disorder, he will simply inspect and then be sure what the matter is. Suffice it to say that the stomach-illuminating apparatus will soon have reached completion; its success seems to be assured. The inventor, a Saxon physician, Dr. Nitsche, has been working now for three years in perfecting his idea, and there seems to be no more doubt that he will be perfectly successful. The principle on which these new instruments are constructed, differs from the old endoscopes in this that the light is not thrown by a reflector from the outside into these cavities, but the light source itself is introduced by the instrument into these cavities, to the very spot intended to be inspected. The light source consists of thin platina wire, made and kept white hot by galvanism. To prevent the instrument from growing warm by the glowing wire, a constant circulation of cold water around the wire is kept up. The arrangement of the water circulation and wire is different, according to the anatomical differences of the several organs, but always so that a perfect and equal cooling of the instrument is produced. In this manner we are enabled to illuminate the different cavities with a degree of intensity that has never been reached before. By use of a special optical apparatus, we are further enabled to considerably enlarge the field of inspection, *i. e.*, it is then possible, through long and narrow tubes, to survey with one glance a large area, as by the use of this apparatus a six to nine centimeter (2½ to 3½ inch) area of the walls of the bladder may be surveyed with the greatest distinctness without moving the instrument.—*Wiener Med. Woche No. 18, May, 1879.*

